# Synthesis and Self‐Assembly of Poly(4‐acetoxystyrene) Cubosomes

**DOI:** 10.1002/marc.202400633

**Published:** 2024-10-16

**Authors:** Marcel Schumacher, Marvin Foith, Manuel Trömer, Nadine Tänzer, Sabine Rosenfeldt, Markus Retsch, André H. Gröschel

**Affiliations:** ^1^ Institute of Physical Chemistry and Center for Soft Nanoscience (SoN) University of Münster Busso‐Peus‐Str. 10 48149 Münster Germany; ^2^ International Graduate School BACCARA University of Münster 48149 Münster Germany; ^3^ Polymer Materials for Energy Storage (PES) Bavarian Center for Battery Technology (BayBatt) and Bavarian Polymer Institute (BPI) University of Bayreuth Universitätsstr. 30 95448 Bayreuth Germany; ^4^ Department of Chemistry Physical Chemistry I and Bavarian Polymer Institute (BPI) University of Bayreuth Universitätsstraße 30 95447 Bayreuth Germany; ^5^ Department of Chemistry Physical Chemistry I Bavarian Polymer Institute Bayreuth Center for Colloids and Interfaces and Bavarian Center for Battery Technology (BayBatt) University of Bayreuth Universitätsstraße 30 95447 Bayreuth Germany

**Keywords:** block copolymers, copolymerization, inverse morphologies, mesoporous microparticles, self‐assembly

## Abstract

Polymer cubosomes (PCs) are a recent class of self‐assembled nanostructures with great application potential due to their high porosity and surface area. Currently, most reported PCs consist of polystyrene block copolymers (BCPs), for which self‐assembly parameters are rather well understood. Changing the block chemistry would be desirable to introduce more functionality; however, knowledge of adapting the self‐assembly process to new chemistries remains limited. This work, reports on synthesizing poly(ethylene oxide)‐*block*‐poly(4‐acetoxystyrene) and its copolymers with styrene, and provide conditions for their self‐assembly into PCs with high yield and high inner order. It is shown that the polarity of the starting solvent toward the corona block allows tuning of the final morphology by controlling the corona volume and packing parameter.

## Graphical abstract

Polymer cubosomes of poly(4‐acetoxystyrene) is realized by RAFT polymerization and subsequent self‐assembly in water. By adjusting the solvent conditions, the same polymer could be directed into polymersomes, cubosomes or hexosomes. The study provides new insights into the formation of polymer cubosomes using functional wall chemistries.

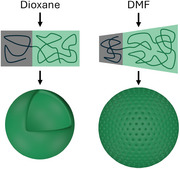

## 1 Introduction

Polymer cubosomes (PCs) and hexosomes are a recent class of mesoporous nanoparticles that can be synthesized from the bottom up by self‐assembly of asymmetric amphiphilic block copolymers (BCPs).^[^ ^1^, ^2^, ^3^, ^4^, ^5^ ^]^ PCs, in particular, are distinguished by a bicontinuous ordered pore structure, where one of the pore systems is closed to the surrounding media while the other one can be accessed from the outside.^[^ ^6^, ^7^, ^8^ ^]^ Therefore, PCs exhibit a large surface area, which makes them of great interest for various applications such as drug delivery,^[^ ^9^ ^]^ energy storage,^[^ ^10^, ^11^, ^12^ ^]^ catalysis,^[^ ^13^, ^14^, ^15^ ^]^ and many more.^[^ ^7^, ^16^, ^17^, ^18^ ^]^

The particle size and shape of PCs are mainly defined by the BCP and the self‐assembly process, which has not been fully clarified yet. During the self‐assembly, a selective solvent (e.g., water), which is only a good solvent for the hydrophilic corona block, is added to a common solvent (e.g., dioxane) in which the BCP is molecularly dissolved. One theory suggests that liquid–liquid phase separation (LLPS) occurs, and polymer‐rich droplets (PRDs) form, which consists of a higher concentration of the common solvent and BCP, while the continuous phase is poor of BCP and enriched with the selective solvent.^[^ ^19^, ^20^, ^21^ ^]^ This process would explain the overall size of PCs that typically reside on the order of micrometers, as larger PRDs should lead to larger PCs.^[^ ^22^ ^]^ The morphology, on the other hand, is mostly predicted by the packing parameter (*P* = *V*/(*a*_0_ *l*_c_)), which provides a good approximation for PC systems as well. It is determined by the ratio of the volume (*V*) occupied by the hydrophobic block to the interfacial area of the chain (*a*_0_) multiplied by the length of the hydrophobic block (*l*_c_).^[^ ^23^ ^]^ While polymers with *P* < 1 are cone‐shaped and therefore assemble into micelles with positive curvature, polymers with *P* > 1 are truncated cones and arranged into inverse morphologies with negative curvature, such as cubosomes and hexosomes.^[^ ^24^ ^]^ Since *P* depends on the block volumes, *P* and therefore the morphology can be changed by block length or through selective swelling/deswelling of domains, e.g. by changing the self‐assembly process or varying the used solvents. So far, solvents have been chosen to address the hydrophobic block to alter its volume and morphology, e.g., it was reported for PEO‐*b*‐PS PCs that dimethylformamide (DMF) is a worse solvent for the hydrophobic block, and the decrease in *l*_c_ led to an increase in *P*.^[^ ^6^, ^25^ ^]^ Unlike lipids, for BCPs *P* not only depends on the volume of the core block but also on the volume of the corona block because the extent of the corona influences interfacial area. Choosing solvents that affect the volume of the corona block could thus as well be a tool to control self‐assembly into cubosomes.

Here, we use reversible addition‐fragmentation chain‐transfer (RAFT) polymerization to synthesize BCPs of poly(ethylene oxide)‐*block*‐poly(4‐acetoxystyrene) (PEO‐*b*‐PA) as well as copolymers with styrene (PEO‐*b*‐P(A‐*co*‐S)) by mixing 4‐acetoxystyrene (A) and styrene (S) in specific ratios. All BCPs were self‐assembled via nanoprecipitation and characterized by scanning electron microscopy (SEM), transmission electron microscopy (TEM) cross‐sections, and small angle X‐ray scattering (SAXS). We demonstrate that the polarity of the common solvent can be chosen such that it primarily affects the dimension of the corona block instead of the core block, overall leading to a reduction of the corona volume during assembly and thus transitions into morphologies with higher *P*.

## 2 Results and Discussion

### 2.1 Polymer Synthesis

**Figure 1:**
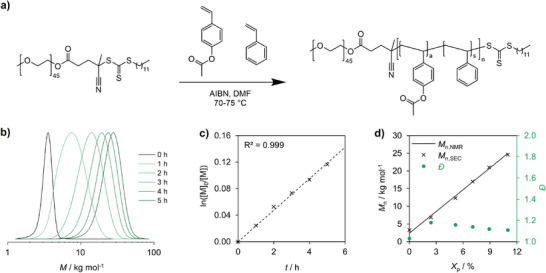
Polymerization kinetics of PEO‐*b*‐PA and PEO‐*b*‐P(A‐*co*‐S) BCPs. a) Reaction scheme of the RAFT copolymerization of PEO‐*b*‐P(A‐*co*‐S). b) SEC measurements in THF (PS calibration) of polymerization kinetics for 100% PA and 0% PS. c) First‐order kinetics of monomer consumption determined by NMR and d) correlation between theoretical *M*_n,NMR_, and measured *M*_n,SEC_, as well as the evolution of *Đ* during polymerization.

All reactions are briefly discussed here (reaction scheme in **Figure** **1**a, all BCP characteristics are summarized in **Table** **1**) and a more detailed description of the polymer synthesis can be found in the experimental section. First, the macro‐RAFT agent was synthesized via Steglich esterification^[^ ^26^ ^]^ using poly(ethylene oxide) methyl ether with a molar mass of *M*_n_ = 2 kg mol^−1^ for the corona block. A functionalization of >99% was determined by nuclear magnetic resonance (NMR) while maintaining a low dispersity of *Đ* = 1.03 determined by size‐exclusion chromatography (SEC). Afterward, the macro‐RAFT agent was used for the polymerization^[^ ^27^ ^]^ of 4‐acetoxystyrene (A)^[^ ^28^ ^]^ and copolymerization with styrene (S) in dimethylformamide (DMF) using azobisisobutyronitrile (AIBN) as radical initiator (Figure 1a). The reaction was monitored via NMR and SEC by taking aliquots every hour. For these exemplary kinetics of A, the curves in the SEC show full initiation, as no peak was observed at the molecular weight of the macro‐RAFT agent (Figure 1b). Additionally, the monomer conversion was determined by NMR. Therefore, the decrease of the vinyl peaks of the monomers was integrated against the signal of the DMF. As such, the degree of polymerization and theoretical number average molar mass (*M*_n,NMR_) could be calculated (Table 1). In addition, it provides information on the copolymerization behavior and ratios of the monomers during copolymerization. The conversion was also used to calculate the logarithm of the monomer concentration at the beginning of the reaction ([*M*_0_]) divided by the monomer concentration at a given time ([*M*]), which was plotted against time (Figure 1c). The high coefficient of determination (0.999) shows first‐order kinetics, as expected for RAFT polymerization. Lastly, the *M*_n,NMR_, and *M*_n,SEC_ were plotted against conversion (*X*_p_) showing almost no deviation from the theoretical values (Figure 1d). The dispersity is comparably high at the beginning of the reaction (*Đ* = 1.18), however, they decrease to *Đ* = 1.10 in the finished BCP (Figure 1b,d).^[^ ^29^, ^30^ ^]^ In summary, all parameters confirm that the polymerization of A (as well as its copolymerization with S) are all well‐controlled, which allows tuning the block length ratio to explore the self‐assembly of PCs. NMR spectra of the purified BCPs are given in Figure S1 (Supporting Information).

**Table 1:** Specifications of different PEO‐*b*‐P(A‐*co*‐C) BCPs.

BCP	*f*_h_ ^a)^ [%]	*M*_n,NMR_ ^b)^ [kg mol^−1^]	*M*_n,SEC_ ^c)^ [kg mol^−1^]	*Đ* ^c^
PEO_45_‐*b*‐PA_138_	92.0	25.0	24.8	1.10
PEO_45_‐*b*‐P(A_0.76_‐*co*‐S_0.24_)_141_	91.5	23.4	20.0	1.18
PEO_45_‐*b*‐P(A_0.57_‐*co*‐S_0.43_)_194_	93.2	29.3	22.0	1.19
PEO_45_‐*b*‐P(A_0.34_‐*co*‐S_0.66_)_228_	93.5	30.8	24.1	1.15
PEO_45_‐*b*‐PS_208_	91.7	24.0	22.4	1.17

^a)^ Hydrophobic block length calculated by NMR;

^b)^ number average molar mass calculated by NMR;

^c)^ number average molar mass and dispersity determined by SEC.

In addition, the reactivity ratios (*r*_S_, *r*_A_) for this system were determined by the Fineman‐Ross method (*r*_S_ = 0.64, *r*_A_ = 1.62).^[^ ^31^ ^]^ Using this information, any desired ratio of A and S can be targeted by adjusting the monomer feed (Figure S2, Supporting Information). Since monomer consumption does not deviate significantly from ideal behavior, a statistical sequence and, thus, a homogeneous monomer distribution along the chain can be estimated. However, A is reacting slightly faster compared to S, which generates a slight overall gradient toward high conversions. We, therefore, stopped all reactions at ≈10% conversion to avoid running into gradient polymers. With the kinetic data from Figure 1, any block length of the hydrophobic block (PA or P(A‐*co*‐S)) can be targeted by stopping the reaction at given times. A library of different BCPs with different ratios of A to S was then synthesized with low dispersity (*Đ* < 1.2) (Table 1) and a hydrophobic block fraction (*f*_h_) in the range where PCs have already been reported for the related PEO‐*b*‐PS system (*f*_h_ > 90%).^[^ ^4^, ^25^, ^32^ ^]^ The obtained molar masses of the BCPs match the expected values determined previously during the kinetics, which further highlights the good level of control for this (co‐)polymerization.

### 2.2 Self‐Assembly and Characterization of the Nanostructures

**Figure 2:**
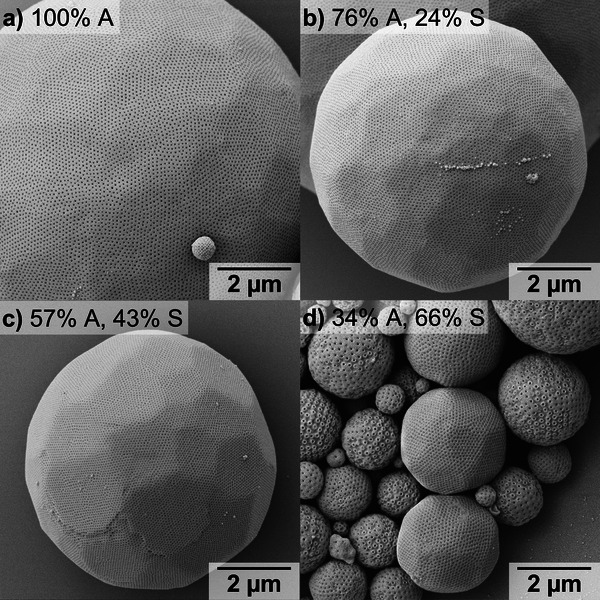
PCs from different PEO‐*b*‐P(A‐*co*‐S) BCPs synthesized via nanoprecipitation going from dioxane (or mixtures of dioxane/DMF) to water. a) PEO_45_‐*b*‐PA_138_ from dioxane. b) PEO_45_‐*b*‐P(A_0.76_‐*co*‐S_0.24_)_141_ from dioxane/DMF 84:16. c) PEO_45_‐*b*‐P(A_0.57_‐*co*‐S_0.43_)_194_ from dioxane/DMF 84:16. d) PEO_45_‐*b*‐P(A_0.34_‐*co*‐S_0.66_)_228_ from dioxane.

Poly(4‐acetoxystyrene) (PA) is an interesting polymer as it can be postmodified to pH‐responsive and hydrogen‐bonding poly(4‐hydroxystyrene) by removing the acetyl group.^[^ ^33^ ^]^ However, it has a slightly higher polarity than styrene,^[^ ^34^ ^]^ and a higher glass transition temperature,^[^ ^35^, ^36^ ^]^ both of which should affect the conditions required for PC formation. Self‐assembly was carried out via nanoprecipitation by dissolving 10 mg of a BCP in 1 mL dioxane or a dioxane/DMF mixture, followed by a dropwise addition of 1 mL of water (selective solvent) within 1 h using a syringe pump while gently stirring the samples. The remaining organic solvent was removed via dialysis against water. By tuning the ratio of dioxane to DMF, PCs with Schwarz D (*Pn3m*) phase have successfully been assembled from all BCPs, which can be seen from the surface pattern of the PCs (**Figure** **2**).^[^ ^19^, ^20^, ^21^ ^]^ Moreover, all PCs exhibit a very homogeneous pore structure, are well‐developed, and are formed in high yield with a negligible amount of side morphologies. The diblock copolymer PEO_45_‐*b*‐PA_138_ with a *f*_h_ = 92% assembled to PCs using dioxane as the starting solvent (Figure 2a). The morphology could be changed, however, by adding DMF to reach a mixture of dioxane/DMF 84:16 (v/v), where PEO_45_‐*b*‐PA_138_ formed polymer hexosomes with a *P6mm* phase instead of PCs (Figure S3a,b, Supporting Information). Polymer hexosomes are typically found at higher *f*_h_ > 95%, which corresponds to an increase in *P*, here realized by the addition of DMF. Upon increasing the S content, PEO_45_‐*b*‐P(A_0.76_‐*co*‐S_0.24_)_141_ and PEO_45_‐*b*‐P(A_0.57_‐*co*‐S_0.43_)_194_ form polymersomes when starting from dioxane instead of PCs, despite suitable *f*_h_ = 91.5% and 93.2% (Figure S3c,d, Supporting Information). The addition of DMF again changes *f*_h_ to reach the PC region due to the increase in *P* and both BCPs form PCs from dioxane/DMF 84:16 (v/v) (Figure 2b,c). A similar trend was reported for PEO‐*b*‐PS PCs, where an increase in DMF content allowed changing the morphology from polymersomes to PCs, because DMF is a less good solvent for PS as compared to dioxane, and the decrease in *l*_c_ led to an increase in *P*.^[^ ^6^, ^25^ ^]^ In our case, the PA block is more polar due to the acetyl ester and should, therefore, be better soluble in DMF.^[^ ^37^ ^]^ Nevertheless, we still observe the same trend. Another reason for the observed changes could be the effect of solvent polarity on the expanded and voluminous corona. The corona block remains always dissolved, and its volume determines the interfacial equilibrium area (*a*_0_), which likewise affects *P*. The main difference is that changes in polarity will have a much bigger impact on the expanded (or contracted) corona as compared to the subtle changes on a collapsed or slightly more collapsed core block. The Hansen solubility parameters support that dioxane is a better solvent for PEO as compared to DMF.^[^ ^38^ ^]^ We performed dynamic light scattering (DLS) measurements of a PEO homopolymer and found a slightly larger hydrodynamic diameter (*d*_h_) in dioxane of ≈4.8 nm as compared to 4.2 nm in DMF (Figure S4, Supporting Information). Consequently, the PEO is more swollen in dioxane and less swollen in DMF. Assuming analogous behavior of the PEO in the corona, larger DMF content decreases PEO volume and *a*_0_, increasing *P*. The solvent polarity will also affect the kinetics of the LLPS, as well as the dimension and composition of the polymer‐rich droplets. The choice of co‐solvents can thus be used to finetune the morphology which we verified for the herein‐reported BCPs, and which should be applicable to other block chemistries as well.

**Figure 3:**
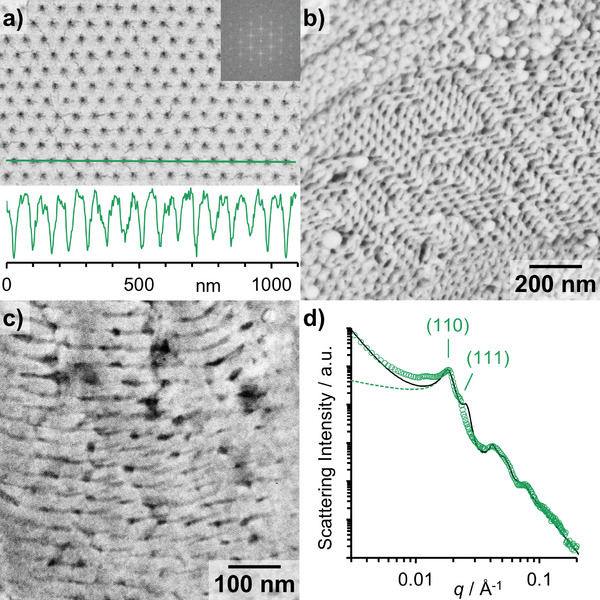
Detailed characterization of PEO‐*b*‐P(A‐*co*‐S). a) SEM image of a PEO_45_‐*b*‐P(A_0.57_‐*co*‐S_0.43_)_194_ PC surface with fast Fourier transform (FFT) insertion in the top right corner and bottom inset grayscale analysis of the green line. b) Inside of a fractured PEO_45_‐*b*‐P(A_0.57_‐*co*‐S_0.43_)_194_ PC analyzed by SEM (identical magnification as in a). c) TEM image of a cross‐section of a PEO_45_‐*b*‐P(A_0.76_‐*co*‐S_0.24_)_141_ PC embedded in resin, cut using an ultramicrotome, and stained with RuO_4_. d) Measured 1D SAXS pattern of PEO_45_‐*b*‐PA_138_ PC powder (green symbols) in comparison with the theoretical scattering intensity of randomly oriented square packed cylinders (green dashed line) with radius *R*, length *L*, and lattice parameter *a* of the P4/mm lattice (*R* = 11.0 ±1.3 nm, *L* = 200 ± 30 nm, both Gaussian distributions, *a* = 34.0 ± 3.4 nm). To account for the scattering contribution due to the micrometer size of the PCs itself a *q* ^−4^ power law was added, resulting in the scattering intensity shown in black. Additionally, the positions of the ratio (√2:√3) of Bragg reflexes of Schwarz D (*Pn3m*) phase are marked, and labeled with the corresponding Miller indices.

Additionally, a more detailed analysis of the PCs was performed (**Figure** **3**). The pore diameter of all PCs with different compositions was evaluated in SEM by grayscale analysis (Figure 3a). The pore diameter was determined to be 13 and 18 nm, regardless of being measured on the surface or inside the particle (Figure 3b). Moreover, all PCs show a similar pore spacing between 68 and 93 nm on the surface and 54–71 nm on the inside of the cracked open particles. This difference could perhaps be because, at the periphery of the particle, the circumference of the pore channels becomes larger. To maintain the ordered periodic structure in the surface grains, the pore spacing on the surface becomes larger. From the fractured PC in Figure 3b, the Schwarz D (*Pn3m*) phase can be clearly seen, especially at the top and bottom left of the image. As additional confirmation, cross‐sections of the particles were prepared and measured by transmission electron microscopy (TEM) (Figure 3c). The mean pore spacing was determined to be 64 ± 5 nm and the mean pore diameter was 14 ± 2 nm, which is in agreement with the results from SEM (67 ± 3 nm; 14 ± 2 nm).

To confirm this result based on mostly surface‐sensitive imaging methods (SEM, TEM), SAXS is used for volume‐sensitive characterization. The SAXS data of a powder of PEO_45_‐*b*‐PA_138_ PCs is shown in Figure 3d. For a *Pn3m* lattice Bragg reflections are expected at *q* positions with the ratio √2:√3 corresponding to the Miller indices (110) and (111).^[^ ^39^ ^]^ Indeed, the corresponding structure peaks are observed in the experimental SAXS intensity, underlining this assumption. The minima (*q*~0.035, 0.067, and 0.094 Å^−1^) of the curve point to an averaged radius of the channels of ≈11 nm. Since PCs consist of a bicontinuous mesophase with two (nearly identical but strongly curved) network channels, modeling the scattering pattern with simple geometric models is out of scope. Nevertheless, in a powder, where the spatial distribution of the PC channels is oriented in all directions, we consider the lattice of randomly oriented square‐packed cylinders (*P4/mm*) as similar enough for a rough comparison. The corresponding theoretical intensity given in Figure 3d is based on cylinders with radius *R* = 11 ± 1.3 nm and length *L* = 200 ± 30 nm, both with Gaussian distributions, and a unit cell dimension (center‐to‐center‐distance of cylinders) of *a* = 34.0 ± 3.4 nm. The attenuation of the first Bragg reflex is an indication of paracrystalline disorder, i.e., disruption of the long‐range order by incorporation of the second channel system. In a good approximation, the unit cell length of the bicontinuous mesophase should then be given by *a*_mesophase_ ≈ 2a = 68 ± 7 nm, which agrees well with the results of SEM and TEM. The deviations between the experimentally observed Bragg reflections (*q*~0.012, 0.018, and 0.023 Å^−1^) and the theoretical intensity are attributed to the presence of interwoven lattices in the mesophase. The scattering of square‐packed cylinders describes the experimental data well for intermediate and higher *q* values but underestimates the forward scattering. As the micron size of the PCs determines the scattering at low *q*, an additional contribution (*q* ^−4^ power law) is added, leading to a good description of the data.

The pore diameter of 22 nm is slightly larger as compared to the value obtained from SEM and TEM (14 ± 2 nm). This can also be explained by the fact that the pore channel is not a cylinder with a constant diameter but can rather be described as a pearl‐necklace structure with pore channels getting larger and smaller periodically. The diameter from imaging was always measured at the narrowest point of the pores, while the diameter from SAXS is to be regarded as an average. The reason only two Bragg peaks can be detected might be due to a polycrystalline lattice or the fact that in some very large particles, there only is a cubosome morphology on the outer layers, but a hexosome morphology in the inner layers of the particle (Figure S5, Supporting Information). As discussed previously, we believe that during self‐assembly the system undergoes LLPS involving the formation of PRDs. Larger PCs originate from larger PRDs where diffusion of the common solvent out of the PRD into the continuous phase takes more time. Thus, the polymers in the core of a PRD also have more time to arrange themselves into a local energetic minimum as the self‐assembly proceeds. Therefore, a hexosome structure can be detected inside the core of some very large particles, while they still feature a cubosome morphology on the outer layers.

## 3 Conclusion

We synthesized a library of different PEO‐*b*‐PA and PEO‐*b*‐P(A‐*co*‐S) BCPs with RAFT polymerization and varied the composition of the second block with different ratios of A to S. By doing so, the polarity and functionality of the polymer could be customized. All polymers have a low dispersity (*Đ* < 1.2), and the targeted molecular weight of the hydrophobic block was in the exact range to form PCs by fine‐tuning the ratio of dioxane to DMF as the common solvent. In addition, polymersomes and hexosomes could likewise be prepared simply by adjusting the ratio of dioxane to DMF for the same BCP. We believe that the common solvent allows shrinking or expansion of the hydrophilic PEO block, thereby tailoring *P*, to obtain the desired nanostructure from polymersome to cubosome to hexosome. These findings may help identify proper self‐assembly conditions for other BCP systems.

## 4 Experimental Section

### Materials

All materials were used as received unless otherwise noted. Poly(ethylene oxide)methyl ether (PEO, *M*_n_ = 2 kg mol^−1^ and *M*_n_ = 20 kg mol^−1^), *N,N’*‐dicyclohexylcarbodiimide (DCC, 99%), styrene (>99%), 4‐cyano‐4‐(((dodecylthio)carbonothioyl)thio)pentanoic acid (RAFT agent, 97%), 4‐dimethylaminopyridine (DMAP, >99%), azobisisobutyronitrile (AIBN, 98%), CDCl_3_ (99.8%) were supplied by Sigma–Adrich. Dichloromethane (DCM) was used from a solvent drying device. 4‐acetoxystyrene (>98%) was purchased from TCI. Dioxane and dimethylformamide (DMF) were obtained from Fisher. Diethyl ether and methanol were technical grades. Water was taken from a Milli‐Q water purification system. The dialysis tubes have a molecular weight cut‐off of 12–14 kg mol^−1^ and were softened in water prior to use.

### Instruments

Size‐exclusion chromatography (SEC) was used to determine the number‐average molecular weight (*M*_n_), molecular weight distribution (*M*_W_, MWD), and dispersity (*Đ* = *M*_W_/*M*_n_). Samples were measured on a 1260 Infinity instrument (Polymer Standard Service, Mainz) equipped with 3 SDV columns (pore sizes 10^4^ Å, 2 × 10^3^ Å) and a SECcurity differential refractometer. HPLC grade THF was used as eluent at a flow rate of 1.0 mL min^−1^ at 40 °C (column oven TCC6000). For each sample, 1 mg was dissolved in 1 mL THF and passed through a 0.2 µm PTFE syringe filter before injecting the sample. A poly(styrene) calibration kit was used to calibrate the system and data was evaluated with the WinGPC UniChrom software.

Nuclear magnetic resonance spectroscopy (NMR) measurements were performed on a Bruker NEO 400. All samples were measured in CDCl_3_ and calibrated using the solvent signal.

*Dynamic light scattering* (*DLS*) was measured using a Zetasizer Nano S from Malvern Panalytical. 10 mg of PEO (*M*_n_ = 20 kg mol^−1^) was dissolved in 1 mL of dioxane or DMF, respectively. The solutions were filtered using a 0.2 µm PTFE filter and transferred to the quartz cuvette with a path length of 10 mm. All samples were measured at 20 °C with an equilibration time of 2 min. All measurements were performed three times with 20–30 runs per measurement.

*Scanning electron microscopy* (*SEM*) was carried out on a Zeiss Crossbeam 340 Dual Beam microscope. 10 µL of the sample solution was put on a clean silicon wafer and dried under reduced pressure. All samples were sputtered with 4.5 nm gold.

*Transmission electron microscopy* (*TEM*). Ultrathin sectioning of the PCs was carried out with a Reichert/Leica Ultracut E microtome (Leica Microsystems, Wetzlar, Germany). The PCs were freeze‐dried, and the resulting powder was embedded in a UV‐curable resin (3D Rapid Resin CLEAR 3DR3582C, wavelength *λ* = 365 nm). The sectioning velocity was ≈1 mm s^−1^, with an inclination angle of 6° to obtain slices of <100 nm thickness. The cross‐sections were transferred to a copper grid. For Ru‐staining, grids were placed in a closed glass chamber containing a small amount of RuCl_3_, which was mixed with circa 0.5 ml of an 11–14% NaOCl solution. The samples were stained for 2 h before the chamber was opened.^[^ ^40^, ^41^ ^]^ Analysis of the cross‐sections was carried out on a TEM (Talos L 120C, Thermo Fisher Scientific) with an acceleration voltage of 120 kV and a LaBF_6_‐filament. The images were taken with a Ceta‐F camera and Velox Software (Version 3.8.80).

*Small angle X‐ray scattering* (*SAXS*) was performed at ambient conditions using a Double Ganesha Air system (SAXSLAB/Xenocs), equipped with a rotating anode X‐ray source (*λ* = 1.54 Å, copper, MicroMax 007HF, Rigaku Corporation, Japan) and a position‐sensitive PILATUS 300K detector (Dectris, Switzerland). The measurements were conducted in 0.6 mm glass capillaries (Hilgenberg, Germany). Different sample‐to‐detector positions were chosen to cover scattering vectors *qϵ*[0.025 Å^−1^, 0.8 Å^−1^]. Radial averaging (intensity *I*(*q*) vs *q*) was performed using the software provided by the instrument. As background, the scattering of an empty capillary was subtracted. The theoretical intensities were calculated using the software Scatter (version 2.5).^[^ ^42^ ^]^

### Methods—Synthesis of the poly(ethylene oxide) macro‐RAFT, PEO‐CTA

For the synthesis of the PEO‐CTA, 2 g of PEO‐OH (*M*_n_ = 2 kg mol^−1^, 1 mmol), 807 mg RAFT agent (2 mmol), and 24.4 mg DMAP (0.2 mmol) were dissolved in 20 mL dry DCM in a round bottom flask. 1032 mg DCC (5 mmol) was dissolved in 20 mL dry DCM in a separate flask. Both flasks were placed in an ice bath and the mixtures were bubbled with argon. The DCC solution was slowly added to the reaction mixture using a syringe. The mixture was stirred from 0 °C to room temperature overnight. Subsequently, the solution was concentrated under reduced pressure, filtrated, and precipitated three times in cold diethyl ether. The product was dried under reduced pressure and a yellow solid was obtained (>99% functionalization (NMR), *Đ* = 1.03 (SEC)).

### Methods—Synthesis of poly(ethylene oxide)‐block‐poly(4‐acetoxystyrene‐co‐styrene), PEO‐b‐P(A‐co‐S)

In a typical reaction, 30.0 mg PEO‐CTA (12.5 µmol), 205 µg AIBN (1.25 µmol), and 31.3 mol monomer were dissolved in DMF. The reaction mixture was purged with argon and stirred at 70 °C or 75 °C depending on the amount of styrene. Subsequently, the reaction mixture was quenched after the desired conversion was achieved. The polymer was precipitated three times in cold methanol and dried under reduced pressure. A white powder was obtained.

### Methods—Self‐Assembly of PEO‐b‐P(A‐co‐S)

For all assemblies, 10 mg of BCP were dissolved in dioxane or a mixture of dioxane and DMF. 1 mL of water was added within 1 h using a syringe pump while stirring gently. Thereafter, the mixture was dialyzed against the water with at least four bath changes. The interval between bath changes was at least 3 h.

## Conflict of Interest

The authors declare no conflict of interest.

## Supporting information



Supporting Information

## Data Availability

The data that support the findings of this study are available from the corresponding author upon reasonable request.
