# Supplementary material for: Synthesis and Self‐Assembly of Poly(4‐acetoxystyrene) Cubosomes
Source: Macromol Rapid Commun. 2024 Oct 16;46(2):2400633. doi: 10.1002/marc.202400633 (PMC11756864; doi:10.1002/marc.202400633)
Supplement: Supplementary file 1 — Supporting Information [file MARC-46-2400633-s001.pdf]

Macromolecular Rapid Communications

Supporting Information

for *Macromol. Rapid Commun.*, DOI 10.1002/marc.202400633

Synthesis and Self-Assembly of Poly(4-acetoxystyrene) Cubosomes

*Marcel Schumacher, Marvin Foith, Manuel Trömer, Nadine Tänzer, Sabine Rosenfeldt, Markus Retsch and André H. Gröschel**

Supporting Information

to

Synthesis and Self-Assembly of Poly(4-acetoxystyrene) and Copolymers into Cubosomes

Marcel Schumacher[1,2], Marvin Foith[3], Manuel Trömer[1], Nadine Tänzer[1], Sabine Rosenfeldt[4], Markus Retsch[4], André H. Gröschel[2,3,]*

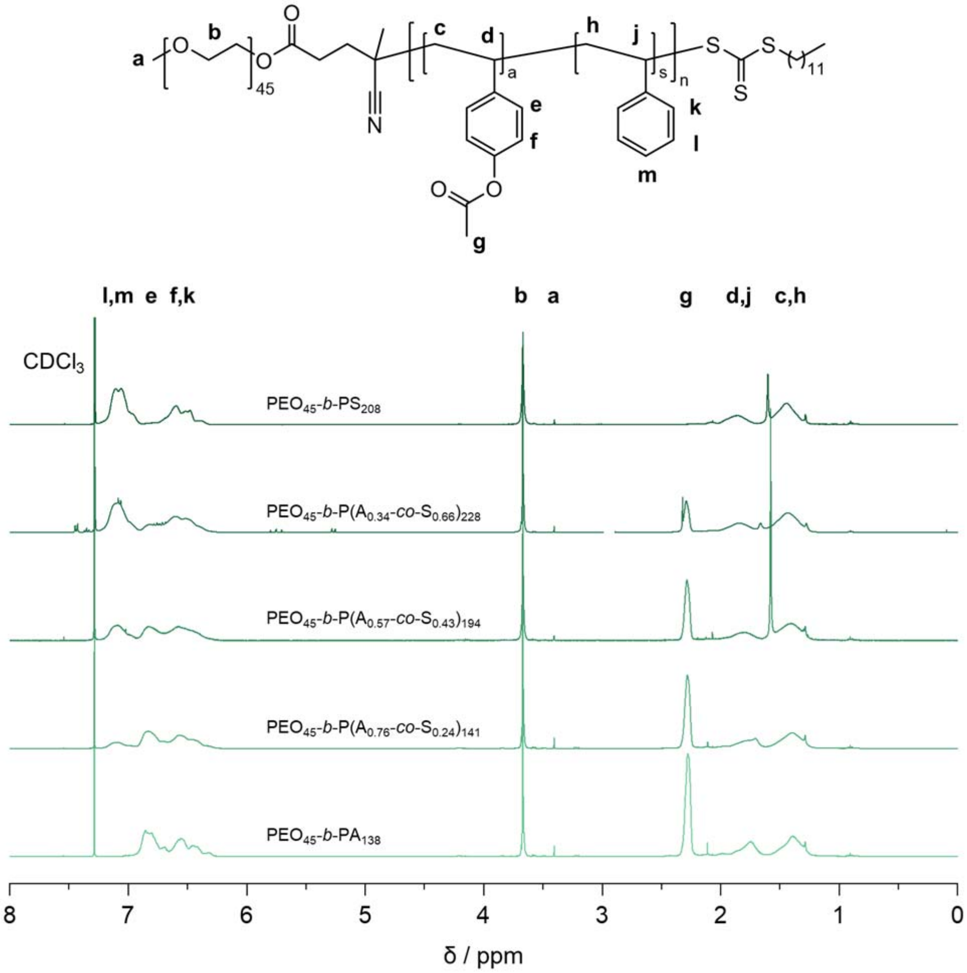

Figure S1. NMR spectra of different PEO-*b*-P(A-*co*-S) polymers normalized by the PEO signal (b).

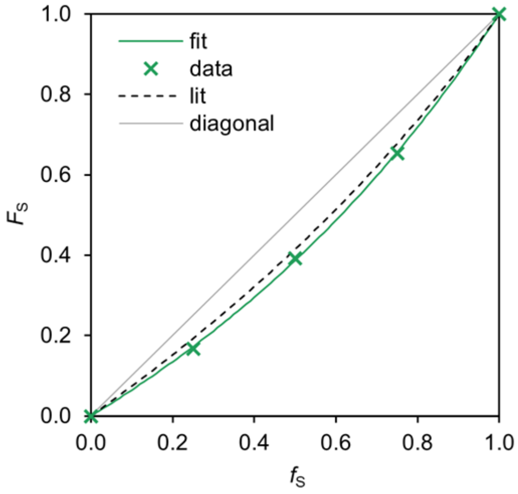

Figure S2. Monomer fraction of styrene (f_S) versus resulting polymer fraction of styrene (F_S) in a PEO-*b*-P(A-*co*-S) block copolymer at low conversion compared to a RAFT copolymerization of A and S from literature.[42]

$$F_S = \frac{r_S f_S^2 + f_S(1-f_S)}{r_S f_S^2 + 2f_S(1-f_S) + r_A(1-f_S)^2}$$

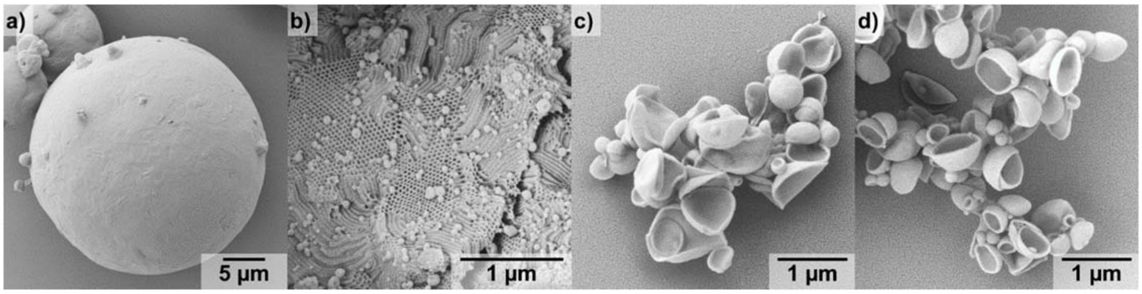

Figure S3. SEM images taken from self-assemblies with different dioxane to DMF ratios. **a, b)** PEO_{45}-*b*-PA_{138} hexosome from dioxane/DMF 84:16 and **(b)** fractured inside. **c)** Polymersomes from dioxane of (c) PEO_{45}-*b*-P($A_{0.76}$-*co*-$S_{0.24}$)$_{141}$ and **(d)** PEO_{45}-*b*-P($A_{0.57}$-*co*-$S_{0.43}$)$_{194}$.

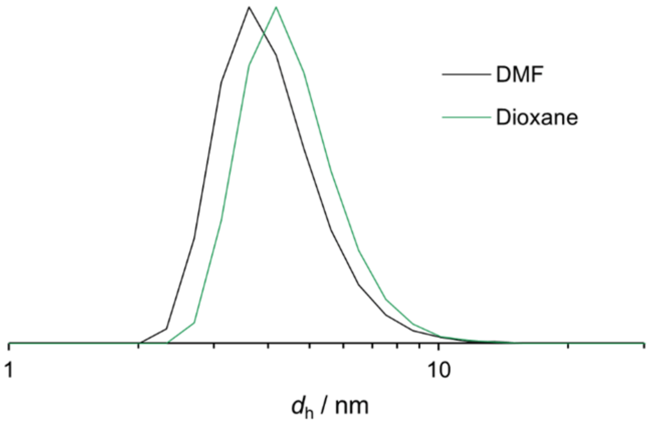

Figure S4. DLS measurements of PEO with a molar mass of 20 kg mol^{-1} in DMF and dioxane.

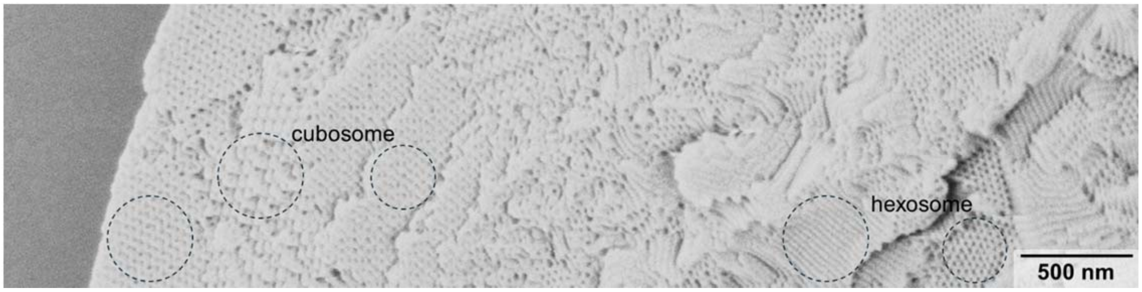

Figure S5. SEM image of a huge particle with cubosome morphology (*Pn3m*) in the outer layer and hexosome morphology (*P6mm*) in the inner layer.
